# Effect of mode of delivery of patient reported outcomes in patients with breast disease: a randomised controlled trial

**DOI:** 10.1097/JS9.0000000000000815

**Published:** 2023-10-04

**Authors:** Eirini Pantiora, Lia-Chasmine Hedman, Iliana Aristokleous, Olivia Sjökvist, Andreas Karakatsanis, Aglaia Schiza

**Affiliations:** a Department for Surgical Sciences, Faculty for Medicine and Pharmacy; b Science of Life Laboratory, Department of Immunology, Genetics and Pathology, Uppsala University; c Department for Plastic Surgery; d Department for Surgery, Breast Surgical Unit; e Department of Oncology, Uppsala University Hospital, Uppsala, Sweden

**Keywords:** electronic patient reported outcomes-measures (ePROs), paper patient reported outcomes-meausres (pPROs), response rate, perceived or confirmed breast disease, randomised controlled trial

## Abstract

**Background:**

Patient reported outcomes (PROs) have an integral role on how to improve patients’ overall experience. The optimal PROs delivery in patients with breast disease is an important issue since PROs are steadily integrated in routine care.

**Methods:**

An institutional phase 3 randomised controlled, open-label trial. Eligible candidates were adult women with perceived or confirmed breast disease. Computer generated randomization was used to allocate interventions: collection of PROs in electronic or paper form. Our objective was the effectiveness of electronic *versus* paper form of PROs. The main outcome measures were: response rate, reported experience, administrative resources, and carbon dioxide emissions.

**Results:**

Two hundred thirty-eight patients were randomised. After loss-to-follow-up and consent withdrawals, 218 participants (median age, IQR=55, 21; *n*=110/*n*=108) were included in the per-intention-to-treat analysis. Response rate was 61.8% for electronic patient reported outcomes (ePROs) and 63.9% for paper patient reported outcomes (pPROs) (difference=−2.1%, 95% CI: −15.8–11.7%). Only known breast cancer at recruitment was predictive for response in multivariable analysis. ePROs were associated with a 57% reduction in administrative time required, a 95% reduction in incremental costs, and 84% reduction in carbon dioxide emissions, all differences being significant. No difference was detected in perception of PRO significance or ease of completion, but participants experienced that they needed less time to complete ePROs [median, (IQR) 10 (9) respectively 15(10)]. Finally, respondents would prefer ePROs over pPROs (difference 48.1%, 95% CI: 32.8–63.4%).

**Conclusion:**

ePROs do not increase the response rate in patients with perceived or confirmed breast disease. However, they can enhance patient experience, reduce incremental costs, facilitate administrative logistics, and are more sustainable. On the basis of these findings, both modalities should continue to be available.

## Introduction

Highlights

- Question: Does the implementation of electronic patient reported outcomes measures (ePROs) instead of paper PROs (pPROs) increase response rates in patients with perceived or confirmed breast disease?
- Findings: In this phase 3 randomιzed controlled trial of 218 participants, the PRO response rate was 61.8% for those who received ePROs and 63.8% for pPROs, a difference that was not significant. However, ePROs decreased administrative costs and facilitated logistics while being preferred by participants.
- Meaning: ePROs resulted in cost and administrative workload reduction and were preferred by the respondents. However, they did not result in an increase of response rates over pPROs in patients with perceived or confirmed breast disease, meaning that both modalities should be offered.

Patient reported outcomes (PROs) have an integral role in the evaluation of quality of life (QoL) both in research and clinical practice, as they reflect the impact of diagnosis and subsequent treatment to patients’ long-term well-being^1,2^. By combining PROs with patient reported experience (PRE), healthcare providers attain valuable information on how to ameliorate patients’ overall experience throughout and after their treatment^3^.

Early breast cancer, with its excellent prognosis that leads to an increasing number of long-term survivors, is a clear paradigm^4^. Furthermore, the diagnosis and surgical treatment of breast cancer stimulate significant changes both on a physical and a psychological level^5,6^. Integration of these parameters can guide future research and clinical practice, allowing for optimization of outcomes beyond mere survival. The latter is particularly significant for breast surgery as, in recent years, the introduction of oncoplastic and reconstructive techniques has allowed for more tailored approaches with the intention of improving survivorship and QoL^7^.

Data for PROs are gathered through standardized and robustly validated questionnaires^8,9^. Questionnaires are traditionally delivered in paper form (pPROs), which patients can answer while waiting in the outpatient clinic or receive via post. Electronic PROs (ePROs) have gradually been introduced in clinical practice with equivalent efficacy^10^. While literature on ePROs for cancer patients is promising^11,12^, there is no data on breast cancer surgery and the data regarding breast surgery focused on cosmetic procedures is scarce^13^. Addressing the issue of optimal PRO delivery in patients with perceived or confirmed breast disease is important, as this is a sizeable subgroup of patients in which PROs are steadily integrated in routine care. The challenges that should be met when assessing PROs and PRE are obtaining high response rates and enhancing patient experience and comfort. Simultaneously, smooth implementation mandates the facilitation of logistics with a sustainable and beneficial resource policy. Despite that the intuitive assumption would be that ePROs may deliver all the above, this has not been tested in a randomised controlled trial (RCT).

The aim of the present trial is to investigate whether a specific delivery mode (ePROs or pPROs) is more advantageous than the other in the context of response rates, PRE, and resources needed in patients with perceived or confirmed breast disease, when routine PRO collection is implemented *de novo*.

## Methods

This institutional parallel, open-label RCT was approved by the Ethical Review Authority in 2019 and conducted in accordance with the Declaration of Helsinki. We obtained written and signed informed consent from all the study participants and the trial was registered at ClinicalTrials.gov. The manuscript was prepared according to the Consolidated Standards of Reporting Trials (CONSORT) Guidelines^14^.

### Study design

An institutional parallel, open-label RCT was undertaken at three departments (Department of Breast Surgery, Department of Breast Radiology, Department of Breast Oncology), between September 2019 and February 2020. Participants were allocated with a 1:1 ratio to receive either pPROs (control group) or ePROs (experimental group). The PROs questionnaires included: BREAST-Q (preoperative or postoperative, as appropriate), Diseases of the Arm, Shoulder, and Hand (DASH) and the European Organization for Research and Treatment of Cancer core and breast-specific modules (EORTC QLQ-C30 and EORTC QLQ-BR23). All questionnaires, including the electronic versions, are validated for use in patients with perceived or confirmed breast disease in our country^15–20^. Following PROs, a questionnaire of Likert items assessed the experience of the participants and their preference.

Eligible candidates were women greater than 18 years old with presumed or confirmed breast disease, attending the Βreast or Οncology outpatient clinics or visiting the Βreast Radiology Department for their scheduled screening. Exclusion criteria were male sex, inability to provide informed consent and linguistic barriers.

Participants enrolling in the study received a pseudonym to fill the PROs. Those allocated to pPROs received the questionnaires via post along with a prestamped envelope for their return. For ePROs, a link was sent via e-mail. The ePRO version was developed in Microsoft Forms (Office 365, Microsoft) and was administered through a safe server. A reminder was sent at 14 and 28 days, via post or e-mail, respectively. Participants who did not respond within a total period of 6 weeks were considered as nonresponders.

Upon response, pPROs were manually processed and the scores were calculated according to the respective instructions and registered in a Microsoft Excel sheet. For the ePROs, the responses were downloaded from the Microsoft Form application in a Microsoft Excel file, and were consequently transferred in another Excel file with macrocommands to automatize score calculation and registration. All these procedures were standardized and timed, following five supervised training sessions.

### Endpoints and statistical analysis

The primary endpoint was to investigate whether the implementation of ePROs could increase the response rate. Secondary endpoints were PRE and patient preference, as assessed by Likert items. Additional secondary endpoints were the administrative time required for the transformation, score calculation, and registration of PROs per arm and monetary costs (including expenses for printing and disseminating PROs and reminders), as a surrogate of the resources required, as well as the respective carbon dioxide emissions (CO_2_e). For the latter, a model was developed in collaboration with the environmental economics group at the Department of Economics of the University of Agricultural Sciences. The carbon emission of an e-mail was calculated at 4 g. Calculating the emissions for the paper PROs has not been equally easy due to multiple factors that must be taken into account (distance, same-day delivery), but data from postal services estimate the minimum carbon emissions of a simple letter at approximately 25 g.

Based on data from current literature and our country’s prospective registry of municipalities and communities, a baseline response rate of 65% was expected^21^. The study was designed to detect significance for a 15% increase in response rate with a Type-I error=0.05 (*P*=0.05) and Type-II error=0.2 (power=80%), resulting in a total sample size of 240 participants. Permuted block randomization with blocks of 8 was performed using the *randomizeR* package, version 1.4.2 (RStudio 3.5.3) and the allocation numbers were sealed in opaque envelopes, stored at the breast clinic. Blinding was not possible, due to the nature of the intervention.

Descriptive statistics were performed. Continuous variables were controlled for normal distribution with the Kolmogorov–Smirnov test and summarized accordingly (mean and SD for normal distribution or median with interquartile range for skewed). Comparisons between study arms were performed by the Student’s *t*-test, the Mann–Whitney test or the independent samples medians test, as appropriate. Categorical variables were presented as numbers and frequencies and the Fisher’s exact test was used for unpaired observations or the McNemar’s test for paired observations. The Wald test was used to assess the difference between proportions. All Likert items were analyzed with nonparametrical tests, as appropriate. Spearman’s Rho test was employed for correlation analyses between the response rate and patient data. If univariate analyses revealed significant associations, subsequent multivariable regression analysis was performed. All statistical analyses were performed in SPSS v28 (IBM) and Stata v16 (StataCorp. 2019. *Stata Statistical Software: Release 16*. College Station LLC.). Statistical analyses were performed primarily per-intention-to-treat, but efficacy analyses were also included, so as to detect differences in the secondary endpoints.

## Results

**Figure 1:**
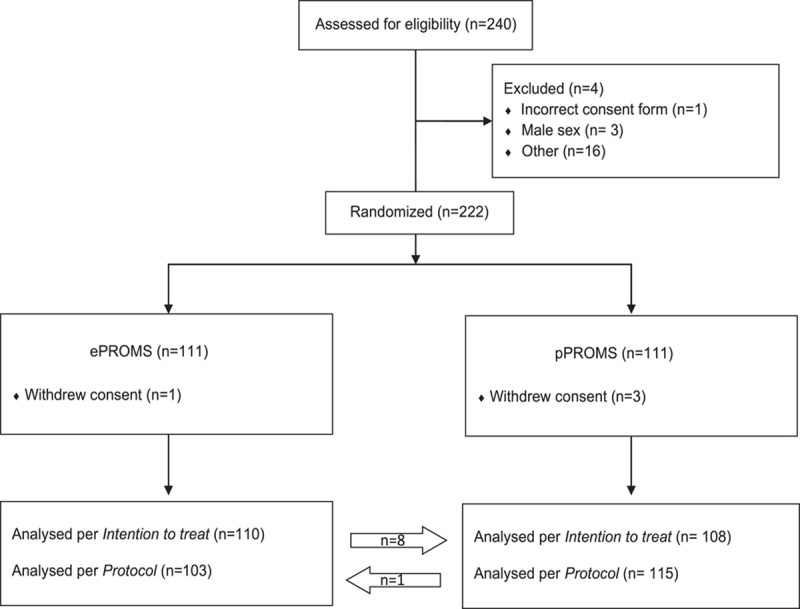
CONSORT diagram of study population; women with perceived or confirmed breast disease. An institutional parallel, open-label randomised controlled trial at three departments at our University Hospital, September 2019–February 2020.

The trial flow is summarized in Figure 1. Postrandomization, four participants withdrew consent, as they experienced that discussing QoL in the context of a recent breast cancer diagnosis or while awaiting screening results was stressful and inappropriate. The period of recruitment was between September 2019 and February 2020. Sixteen enrolled in the study just prior to the SARS-2-pandemic. While several had responded spontaneously within the trial timeframe, no reminders were sent to nonresponders due to circumstance and investigators chose to exclude this group from further analyses. This left a total of 110 participants in the ePROs arm and 108 in the pPROs arm for the per-intention-to-treat analysis. Eight participants from the ePROs arm chose to receive pPROs and one vice versa (difference 3.2%, 95% CI: 0.09–6.3%; Mc Nemar’s test, *P*=0.039). Interestingly, post-tolerance of participant preference, the median age in the ePROs group was younger than the pPROs group (52 vs. 59 years, *P*=0.006). The characteristics of the study population are presented in Table 1.

**Table 1:** The characteristics of the study population per-intention-to-treat and per-protocol analyses.

	Per intention-to-treat			Per-protocol		
	ePROs	pPROs		ePROs	pPROs	
Age (years)^a^	53.5 (22)	57 (19)	0.091^b^	52 (22)	59 (19)	0.006^b^
Age (years)^c^	53.3 (15.1)	57.1 (13)	0.051	52.1 (14.9)	58 (13.1)	0.002^d^
Site of recruitment^e^						
Breast surgery	68 (61.8)	66 (61.1)	1.000^f^	64 (62.1)	70 (60.9)	0.969^f^
Oncology department	4 (3.6)	4 (3.7)		4 (3.9)	4 (3.5)	
Radiology department	38 (34.5)	38 (35.2)		35 (34.0)	41 (35.7)	
Known breast cancer at enrollment^e^						
Yes	33 (30.0)	33 (30.6)	1.000^f^	31 (30.1)	35 (30.4)	1.000^f^
No	77 (70.0)	75 (69.4)		72 (69.9)	80 (69.6)	
Previous breast surgery^e^						
None	77 (70.0)	75 (69.4)	0.975	72 (69.9)	80 (69.6)	0.879
BCT	25 (22.7)	24 (22.2)		24 (23.3)	25 (21.7)	
Mastectomy	8 (7.3)	9 (8.3)		7 (6.8)	10 (8.7)	

One-third of the patients were diagnosed with breast cancer.

a Median, (interquartile range).

b Mann–Whitney test.

c Mean (SD).

d Student’s *t*-test.

e *n*, (%).

f Exact Fischer’s test.

BCT, breast conserving therapy, ePROs, electronic patient reported outcomes forms, pPROs, paper patient reported outcomes forms.

The overall response rate was 62.8% (95% CI: 56.2–69.5%). In per-intention-to-treat analysis, ePROs did not increase response rates over pPROs (61.8 vs. 63.9%; difference=−2.1%, 95% CI: −15.8–11.7%, Wald test *P*=0.860). Efficacy analysis did not differ; response rates were 62.1% for ePROs and 63.5% for pPROs (difference=−1.3%, 95% CI: −15.1–12.4, Wald test *P*=0.949). Response rates had a significant increase after both the first reminder (45.9 vs. 57.8%, difference 11.9%, 95% CI: 5.4–18.4%; Mc Nemar’s test *P*<0.001), and the second, albeit not so accentuated (57.8 vs. 62.8%, difference 5.0%, 95% CI: 1.7–8.4%, McNemar’s test *P*=0.001). Mean participant age (SD) was older among responders [56.7 (13.9) vs. 52.5 (14.4); mean difference 4.3, 95% CI: 0.3–8.3, Student’s *t*-test *P*=0.017). Moreover, known breast cancer at the time of recruitment (patient vs. healthy participant) also increased response rates in univariate analyses (77.3 vs. 56.6%; difference 20.7%, 95% CI: 7.9–33.5, Wald test *P*=0.004). However, only the latter retained significance in multivariable regression analysis (Table 2).

**Table 2:** Factors affecting response frequency.

	Univariate analysis			Multivariable analysis	
	Response	No response	*P*	Exp(B), 95% CI	
Age (years)^a^	53 (20)	58 (20)	0.049^*^	1.005 (0.979, 1.032)	0.722
Age (years)^b^	52.5 (14.4)	56.8 (13.9)	0.034^**^		
Known breast cancer at enrollment^c^					
Yes	51 (77.3)	15 (22.7)	0.004^d^	3.009 (1.304, 6.941)	0.010
No	86 (56.6)	66 (43.4)			
Reminders^c^					
Yes	48 (37.2)	81 (62.8)	<0.001^d^	0.000	0.996
No	89 (100.0)	0 (0.0)			

This table represent the profile of the responders; showing that only breast cancer diagnosis retained significance in multivariable regression analysis.

a Median, (interquartile range).

b Mean (SD).

c *n*, (%).

d Exact Fischer’s test, Exp(B): Exponentiated B coefficient.

* Mann–Whitney test.

** Student’s *t*-test.

Experience reported outcomes are presented in Table 3. The participant response was equally positive between arms about the importance of PROs and their own participation to trials. At the same time, respondents judged that it took less time to fill the ePROs than the pPROs. ePROs were deemed more ʻuser-friendlyʼ, but the difference was not significant. However, 91.2% of those who were allocated to ePROs stated that they would prefer the electronic PROs as the standard method of delivery, whereas the respective preference for pPROs was 56.9% (difference 48.1%, 95% CI: 32.8–63.4%, Wald test *P*<0.001). Analyses per-intention-to-treat and per-protocol did not reveal any differences in outcomes.

**Table 3:** Participant reported experience outcomes per-intention-to-treat and per-protocol.

			
	ePROs	pPROs	*P*
a. Per intention to treat			
‘Perception on PRO Importance and Attitude towards participation’^†^	5 (1)	5 (1)	0.358^δ^
‘User friendly’^†^	5 (1)	4 (2)	0.388^δ^
Time required (min)^†^	10 (9)	15 (10)	<0.001^*^
Preferred format for ‘standard use’^‡^			
ePROs	62 (91.2%)	28 (43.1%)	<0.001^ll^
pPROs	6 (8.8%)	37 (56.9%)	
b. Per-protocol			
‘Perception on PRO Importance and Attitude towards participation’^†^	5 (1)	5 (1)	0.796^δ^
‘User friendly’^†^	5 (1)	4 (2)	0.120^δ^
Time required (min)^†^	10 (9)	15 (10)	0.002^*^
Preferred format for ‘standard use’^‡^			
ePROs	63 (98.4%)	27 (39.1%)	<0.001^ll^
pPROs	1 (1.6%)	42 (60.9%)	

The vast majority of those who were allocated to ePROs prefer the electronic PROs as standard method of delivery.

* Mann–Whitney test.

† Median, (interquartile range).

‡ *n*, (%).

δ Independent medians test.

ll Exact Fischer’s test, PROs, Patient reported outcomes, e, electronic, p, paper.

When analyzing the administrative time and the cost required for processing PROs inquiries for each patient, the ePROs reduced administrative time by 57 and 95% incremental cost reduction, primarily through material savings in stationary and administrative savings in postal expenses. Finally, CO_2_e through the routine implementation of ePROs was reduced by 84%. These outcomes were not affected by the number of reminders, which did not differ between the study arms. The results are presented in Table 4.

**Table 4:** The ePROs reduced the administrative time required for the registration of PROs as well as monetary costs and carbon dioxide emissions.

			
	ePROs	pPROs	*P*
a. Per intention to treat			
Administrative time per patient (min)^a^	3.8 (1.70)	8.9 (1.70)	<0.001^**^
Monetary cost per patient (€)^a^	0.01 (0.05)	0.20 (0.07)	<0.001^**^
CO_2_e (gr)^b^	12 (4)	75 (25)	<0.001^*^
b. Per-protocol			
Administrative time per patient (min)^a^	3.4 (1.03)	8.9 (1.59)	<0.001^**^
Monetary cost per patient (€)^a^	0.00 (0.00)	0.20 (0.07)	<0.001^**^
CO_2_e (gr)^b^	12 (4)	75 (25)	<0.001^*^

a Mean, (SD).

b Median, (interquartile range).

* Mann–Whitney test. Min: minutes.

** Student’s *t*-test.

€, Euros, CO_2_e, Carbon dioxide emission, gr, grams.

## Discussion

The present RCT did not confirm the hypothesis that ePROs can *per se* increase the response rate in patients with perceived or confirmed breast disease. At the same time, ePROs were more popular among respondents and their implementation came with significant cost and resource containment as well as a significant reduction of CO2e. Similar research in orthopedics and hand surgery have shown contradictory results with regards to response rates. In these studies, ePROs had the lowest response rate^22–24^. These studies, however, addressed a different patient population and different medical conditions. In any case, it becomes evident that implementing ePROs only is not a ripe strategy to maximize response rates. The availability of different modes of delivery, flexibility in implementation and attention to patient preference may address this conundrum. It is expected though, that people will become more and more familiar with ʻe-healthʼ as a consequence of technological development. Therefore, ePROs prevalence would probably not come as a surprise.

Stimulating response was found to be challenging and independent of mode of delivery. While, when preference was tolerated, the mean age of those that chose ePROs was younger, this did not translate in a respective increase in response rates. And, despite that one would expect that an older population might be less familiar with the internet or e-mail, univariate analysis suggested that the mean age was older among responders. Additionally, ePROs were more popular compared to pPROs among responders, but that was not accompanied by a difference in response rates, either. Reminders, seemed to increase response, a finding in line with previous studies on oncologic patients^25,26^, but this effect was again not retained on logistic regression. Therefore, it may be so that familiarity and ease of implementation are not sufficient triggers for a response. The only factor that unequivocally increased response rates was an already established breast cancer diagnosis at the time of trial participation. This is an important finding, as it suggests that patients, in contrast to healthy participants, are much more aware of the importance of their feedback on QoL bares. The most plausible interpretation is that, beyond factors that have been discussed in the literature, the perception that response and information provision may facilitate monitoring or motivate intervention from the healthcare providers, may yield a benefit on the individual level, which is most often not the case for healthy participants, that might have viewed this trial as a mere survey. The trial results suggest that the ease and flexibility of ePROs need to be taken into consideration when routine implementation is intended. However, both modes of delivery should be available, at least until more factors that trigger response or optimization pathways in ePRO delivery have been established.

The environmental impact of the two methods is also an aspect that needs to be commented. Ensuring sustainable methods of healthcare implementation and follow-up is becoming increasingly relevant in the face of the challenges posed by climate change. The finding that large-scale implementation of PROs would lead to a substantial difference in carbon emissions between the two methods in favor of ePROs, is important information for all the involved stakeholders that could motivate the promotion and refinement of this modality.

From a pragmatic standpoint, despite this being a single center trial, it was specifically designed and conducted in a unit that meant to implement PROs without pre-existing resources or routines, with the ambition to emulate the circumstance of *de novo* PROs implementation, seeking the simplest and most cost-efficient approach. Interestingly, while none of the three departments that included patients in this study had an existing routine of PRO collection, the implementation was extremely uncomplicated and costless. The instruments used are easily accessible and available for clinical use and research, as well as validated for many different languages. The Microsoft Forms application is included in a software (Office), which is freely available in most units. An additional advantage of this approach was that, while standardised PROs sheets do not provide space for comments from patients, in a questionnaire formulated in Microsoft forms it was possible to include a free-text module where patients could provide their own thoughts and concerns, allowing for more flexibility and tailored-follow-up. The design and execution of this study shows that the routine implementation of PROs is feasible and realistic in comparable clinical settings.

A common restriction in these studies is the inability to evaluate ʻnonresponder biasʼ^27^. Despite that no other factors other than a known breast cancer diagnosis was identified, there were participants who felt that having to answer these questionnaires while under investigation of a breast lesion or waiting screening results was challenging. Feedback from participants who withdrew consent or had a negative attitude towards the study showed that they found it inconsiderate to be involved in this study while being in such a stressful position. This is in line with current literature, which shows that the shock of cancer diagnosis affects patient perception of their quality of life, leading to inaccurate assumptions^6,28^. On the contrary, retrospective collection of baseline PROs has proven much more effective with regards to that, while concerns for ʻrecall biasʼ seem to be negligible^29^.

Regardless of the method used, the importance of regularly assessing and evaluating the QoL parameters of patients with breast cancer has been discussed extensively and many studies have contributed towards a way of accumulating data that is more standardised and more effective for all involved. Indicative of this is a review from Howell *et al*.^30^, on studies evaluating quality of life in oncologic patients, which showed that most studies were focused on breast cancer patients.

## Conclusion

Our findings indicate that at present, both modalities of delivery should continue to be available. Although ePROs may enhance patient experience, reduce incremental costs, facilitate administrative logistics and are more sustainable compared to pPROs, their implementation did not increase response rate in the examined population. It is important to implement universally applicable PROs as well as PREs not only in research settings but also in routine care, as it results in optimal, patient-centered care and value-based care for patients with both confirmed or perceived disease.

## Ethical approval

This institutional parallel, open-label RCT (Approval number: 2019-05740) was approved by the Swedish Ethical Review Authority, Uppsala University Hospital, Uppsala County in 2019.

## Consent

Written informed consent was obtained from the patient for publication and any accompanying images. A copy of the written consent is available for review by the Editor-in-Chief of this journal on request.

## Sources of funding

No funding.

## Author contribution

E.P.: data curation, formal analysis, investigation, project administration, software, visualization, and writing – original draft; L.C.H.: formal analysis, visualization, writing –original draft; I.A. and O.S.: investigation; A.K.: conceptualization, formal analysis, investigation, methodology, project administration, resources, software, supervision, visualization, writing – review and editing; A.S.: investigation, project administration, visualization, writing – review and editing.

## Conflicts of interest disclosure

The authors declared no potential conflicts of interest with respect to the research, authorship, and/or publication of this article.

## Research registration unique identifying number (UIN)

1. Name of the registry: https://www.clinicaltrials.gov/
2. Unique identifying number or registration ID: NCT04718324.
3. Hyperlink to your specific registration (must be publicly accessible and will be checked): https://clinicaltrials.gov/ct2/show/NCT04718324

## Guarantor

Associate Prof A.K. and PhD A.S. accept full responsibility for the work. Associate Prof A.K. had full access to all the data in the study and takes responsibility for the integrity of the data and the accuracy of the data analysis.

## Availability of data and materials

The datasets used and/or analyzed during the current study are available from the corresponding author on reasonable request.

## Presentation

Preliminary partial results from the study were presented at 40th ESSO, Portugal, 9th November 2021.
